# Fabrication of PCL/CMARX/GO Composite Nanofibrous Mats for Dye Adsorption: Wastewater Treatment

**DOI:** 10.3390/membranes13070622

**Published:** 2023-06-26

**Authors:** Mona Saad Binkadem

**Affiliations:** Department of Chemistry, College of Science, University of Jeddah, P.O. Box 80327, Jeddah 21589, Saudi Arabia; 04100507@uj.edu.sa

**Keywords:** adsorption, bacterial cellulose, electrospinning, thin nanofibrous mat, wastewater treatment, environmental pollution treatment

## Abstract

The effluents of industrial wastewater contain several toxic organic and inorganic pollutants that may contaminate clean and freshwater sources if untreated or poorly treated. These toxic pollutants include colors; hazardous compounds; surfactants; cosmetics; agrochemicals; pharmaceutical by-products; and agricultural, pharmaceutical, and medical contaminants. Treating wastewater has become a global problem. Many projects have been started in the last two decades to treat wastewater, resultant water pollution, and associated waste management problems. Adsorbants based on graphene oxide (GO) are viable wastewater treatment materials due to their adaptability, photocatalytic action, and capacity for self-assembly. Here, we report the fabrication of nanofibrous mats from polycaprolactone (PCL), carboxymethyl arabinoxylan (CMARX), and carboxyl-*functionalized*-graphene oxide using an electrospinning technique. The silver nanoparticles were loaded onto the mat to enhance their photocatalytic activity. These mats were characterized using different techniques, including Fourier transform infrared (FTIR), scanning electron microscope (SEM), and transmission electron microscope (TEM). The water contact angles were used to study their hydrophilic and hydrophobic behavior. The Langmuir isotherm model and adsorption kinetics were studied to evaluate their adsorption capabilities against methylene blue (MB). Sample 2 followed the Langmuir isotherm model (R^2^ = 0.9939). Adsorption kinetics exhibited pseudo-second order behavior (R^2^ = 0.9978) due to their maximum correlation coefficient values. MB has excellent adsorption at room temperature and the formation of the monolayer at the surface of the adsorption mat. An enhanced PO_4_^3−^ and MB adsorption was observed, providing recyclability up to 4–5 times. Hence, the fabricated nanofibrous mat would be a potential candidate for more effective wastewater treatment applications.

## 1. Introduction

Modern industrialization has developed all the necessary products to facilitate the increasing population. The effluent from the wastewater often remains untreated or poorly treated, causing increased water pollution and contaminating freshwater resources. The rapid expansion of the mining, paper, and battery industries, as well as agriculture, medical, and food industries, have contributed to water pollution due to their inorganic and organic contaminants. Recent industrial growth has resulted in heavy metals, hazardous substances, colors, and pharmaceutical waste accumulating in water bodies [1,2]. The water bodies contaminated by dye effluent contain suspended solids, COD, dye, and other toxic chemicals [3,4]. Therefore, the treatment and recycling of wastewater require the use of cutting-edge materials and methods. The materials require high absorbency, variable permeability, commercial viability, and recyclable qualities. Since the development of electrospun nanofibers, their potential benefits have spread to several academic disciplines [5,6]. The electrospun technique has received much attention as a novel way to make polymer nanofibers because it is straightforward to scale up and has a high production rate with ideal characteristics [7]. Nanofibers eventually lead to the creation of a membrane with a variety of properties. It is preferable to have high values for specific surface area, pore volume, size tunability, and permeability. It is a perfect candidate for membrane-based water purification filters [8]. Various nanocomposite nanofibrous membranes and nanomaterials were used in their fabrication to improve the structural and functional properties.

Natural polymers have drawn increasing attention because of their distinctive qualities, which include ease of chemical functionalization, stability in both heat and cold, and fouling resistance. Thus, various contaminants with biosafety were studied for water contaminant removal using natural polymers [9]. Natural polymers, including chitosan [10,11,12], guar gum [13,14,15], arabinoxylan [16,17], cellulose [18,19], and alginate [20,21,22], have been reported in several applications, especially in water treatment, due to their biocompatible and biosafety characteristics. They have been extensively studied in the field of water treatment. Similarly, various synthetic polymers, such as polyvinyl alcohol (PVA) [23,24], poly(-caprolactone) (PCL) [25], poly(lactic acid) (PLA) [26], and others [27,28], have been reported in the development of composite materials for different applications, including medical and water. These mixtures can improve the surface area, fiber thickness (which helps nanoparticles anchor to nanofibers), and pore dispersion of wastewater treatment systems. Since titanium oxide (TiO_2_) nanoparticles were incorporated into nanofibers to produce reactive oxygen species (ROS) that will aid in degrading dyes and other toxins, removal activities have improved [29].

Graphene oxide (GO) is known due to its excellent physicochemical characteristics. It is a 2D material and can easily functionalize polymeric nanofibrous in small quantities [30]. The permeability and wettability of the novel composite materials for medical applications, including wastewater treatment, can be greatly improved by adding various hydrophilic functional groups [31,32]. These oxygen-based functional groups produce ROS that facilitates dye degradation and other colorizing toxins. Silver nanoparticle@graphene oxide (Ag@GO) composites are currently thought to be a potential candidate for resolving this limitation naturally. The large surface area and oxygen-based functional groups can easily enable the electrostatic immobilization of Ag nanoparticles [33]. However, silver nanoparticles that anchor on GO sheets may aggregate, greatly reducing their ability to disperse in organic solvents and water due to the GO’s ability to control the wettability and permeability of nanofibrous membranes when incorporated into a polymeric matrix [33]. It also can cause immiscibility with the polymeric matrix that may result in poor structural, functional, morphological, and other desirable parameters that may affect the performance of the polymeric membrane during filtration [33].

In this study, we have developed a composite nanofibrous mat; the nanofibrous mat was fabricated by electrospinning technique using PCL/CMARX/GO-*f*-COOH (Sample 1/PCG) with standard operating protocols and parameters. The PLA/CMARX/GO-*f*-COOH was thermally processed, and AgNO_3_ was used to anchor over the nanofibrous mat as PCL/CMARX/GO-*f*-COOH@Ag (Sample 2/PCG@Ag). The recently developed composite nanofibrous mats have desirable physicochemical and mechanical stability. Treating wastewater by removing toxic and unwanted materials is cost-effective, and newly developed nanofibrous materials have never been reported. The manufactured nanofibrous mats are cost-effective, environmentally friendly, and may be used to treat wastewater.

## 2. Materials and Methods

### 2.1. Materials

*Plantago ovata* seed husks were bought from the local market of Johor Bahru, Malaysia, to extract arabinoxylan (ARX), graphene oxide (GO), sodium mono-chloroacetate (ClCH_2_COONa), sodium hydroxide (NaOH), polycaprolactone (PCL), and silver nitrate (AgNO_3_). Chloroacetic acid (CH_3_COOCl), Rhodamine B (Rh-B), and methylene blue (MB) were supplied by Sigma-Aldrich, Selangor Malaysia. The hydrogen peroxide (H_2_O_2_) and hydrochloric acid (HCl) were purchased from Aladdin Reagent, Shanghai, China.

### 2.2. Methods

#### 2.2.1. Synthesis of Carboxymethyl–Arabinoxylan

Arabinoxylan is a natural polymer, and it is extracted from the seed husk of psyllium using a reported procedure with little modification [34]. A total of 500 g of *Plantago ovata* seed husk was soaked overnight in 2.5 L deionized water. Then, it was blended with NaOH solution (2.5%) for 5 min, and vacuum filtration separated the insoluble husk particles from gelation. The gelling part was treated with acetic acid to coagulate. The obtained gel was washed with plenty of deionized water to neutralize, and the obtained gel was freeze-dried to obtain dried ARX powder. Then, 2 g ARX was placed into ethanol for 1 h with continuous stirring at room temperature by adding ClCH_2_COONa and NaOH (20%) solution. The reaction mixture was allowed to be stirred at 55 °C for 5 h to synthesize the CMARX. After 5 h, the reaction media was washed with an 80% water/methanol mixture (*v*/*v*) to obtain CMARX. The synthesized CMARX was treated with acetic acid to neutralize and was washed with ethanol. It was dried in a vacuum oven to obtain a fine powder of CMARX.

#### 2.2.2. Matrix Solution Preparation

The graphene oxide was functionalized with carboxyl (GO-*f*-COOH) and freeze-dried at −50 °C [35]. The solutions of PCL (15 weight %) and CMARX (25 weight %) were separately prepared in deionized water. The solutions were prepared with controlled gelation to fabricate a nanofibrous mat. The homogenized graphene oxide solution functionalizes carboxyl, and CMARX was prepared at 45 °C with continuous stirring for 2 h. It was added to the PLA solution with a 1:1 ratio and again stirred for 2 h to obtain a homogenized solution. These solutions were used as a polymeric matrix to fabricate nanofibers via electrospinning.

#### 2.2.3. Fabrication of Nanofibrous Electrospun Mat

The prepared solutions were filled in the syringe and fixed with a stain-free steel needle (diameter of 0.6 mm). The constant flow rate of solution from the syringe was adjusted to 0.25 mL/h and operated under a strong voltage difference of 20 kV. The needle and collector were separated by 25 cm, and the collector was covered with aluminum foil to collect the resultant nanofibers. After collection, the nanofibers were gathered on aluminum foil and dried in the oven. These nanofibers were oven-dried at 150 °C for 2 h for heat-induced crosslinking between −OH and −COOH through esterification. Therefore, PCL ensured the structural integrity, CMARX provided desirable gelation, and GO enhanced the functional behavior via π–π stacking. Thanks to the carboxylic functional groups, the silver nanoparticles were in a very bad environment. The combined synergistic catalytic effect of these individual behaviors has produced wastewater treatment benefits for dye degradation.

#### 2.2.4. Preparation of Composite Nanofibrous Mat

The nanofibrous mats were dipped into ascorbic (1 mL) acid-added silver nitrate solution (30 mg/mL), and the reduced silver nanoparticles initiated their anchoring onto the nanofibrous mat. The dipping of nanofibrous mats was repeated after different intervals (1, 2, 3 h) to anchor maximum silver nanoparticles. These silver nanoparticles were removed from the nanofibers by washing them in deionized water after 3 h. These nanofibrous mats were dried at 85 °C to prepare a dried nanofibrous mat for wastewater treatment.

## 3. Characterizations

The functional groups and structural analysis of nanofibrous mats were conducted using Fourier transform infrared spectroscopy (Shimadzu FTIR-8100A, Kyoto, Japan). The FTIR analysis was conducted from a 4000 to 400 cm^−1^ wavenumber. The surface morphology was analyzed using scanning electron microscopy (JEOL-JSM 5410 LV) with an accelerated voltage of 10 kV. The nanofibrous mats were dried well and gold-sputtered before analysis. The aggregates of silver nanoparticles were observed through the transmission electron microscope (HT7700, Hitachi, Japan). A UV–Vis spectrophotometer (HATCH D500, Boston, MA, USA) was used to observe dye adsorption.

### Adsorption Studies

The various concentrations of methylene blue (MB), a reference dye used to study wastewater treatment, were examined using a UV–Vis spectrophotometer at 664 nm. A known concentration of a standard solution was used to calibrate the UV–Vis spectrophotometer. The recently developed composite nanofiber mat was present during the batch-mode adsorption studies of MB. Similar research has been carried out on the effects of MB concentrations (200–1000 mg/L) on adsorption efficiency. Under optimal conditions, a constant concentration of (1 mM) of inorganic anions (Cl^−^, SO_4_^2−^, HCO_3_^−^, and PO_4_^3−^) was used to study their impact on the effectiveness of MB removal. Composite nanofibrous mats were tested for their ability to be recycled using a diluted HCL solution (2 mM) at an ambient temperature for 5 h. The nanofiber mat’s surface charge then inverts from negative to positive, allowing MB to be removed from the adsorbent surface via electrostatic repulsion. Five cycles of regeneration were tested using the same methodology. The composite nanofibrous mats were washed twice with water for the subsequent adsorption cycle. The concentration of MB was once more measured to determine the adsorption capacity of composite nanofibrous mats.

## 4. Results and Discussion

### 4.1. FTIR Analysis

**Figure 1:**
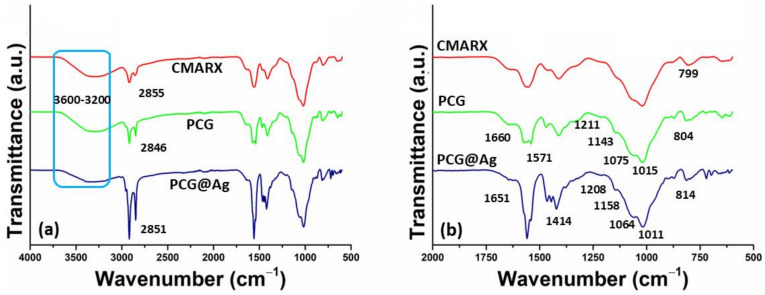
FTIR analysis of the (PCL, CMARX) fabricated nanofibrous mat and (Sample 1 and Sample 2) composite nanofibrous mat. (**a**) is the FTIR profile from 4000 to 500 cm^−1^ and (**b**) 2000 to 500 cm^−1^, respectively.

The functional groups and structural behavior of fibrous mats were presented using FTIR spectral profile, as shown in Figure 1. The characteristic peaks of PCL at 1143, 1211, 1294, 2851, and 2923 cm^−1^ are due to C=O–C (stretching), C–O–C (asymmetric stretching), C–O, C–C (stretching), –CH_2_ (symmetric and asymmetric) vibrations, respectively [36]. The vibration peak at 1660 cm^−1^ is due to carboxyl –C=O (stretching). The vibration peak at 1651 cm^−1^ is due to C=C stretching vibration. The graphene oxide characteristics peaks of oxygen-based functional groups at 1552, 1414, and 1374 cm^−1^ are attributed to functional groups C=O (stretching), C–O (stretching), and C=O (stretching) vibrations, respectively [21,37]. The broader absorption peaks 1178-948 cm^−1^ and 919 cm^−1^ are due to saccharine structure and pyranose ring, as these confirm the presence of CMARX [38]. The broadband at 3600-3200 cm^−1^ is characteristic of O–H stretching absorption and describes intramolecular and intermolecular hydrogen bonding. The FTIR analysis of the PLA has been provided in the Supplementary Figure S1. However, the XRD analysis of the Sample-1 and Sample-2 has been provided in the Supplementary Figures S2 and S3.

### 4.2. SEM Analysis

**Figure 2:**
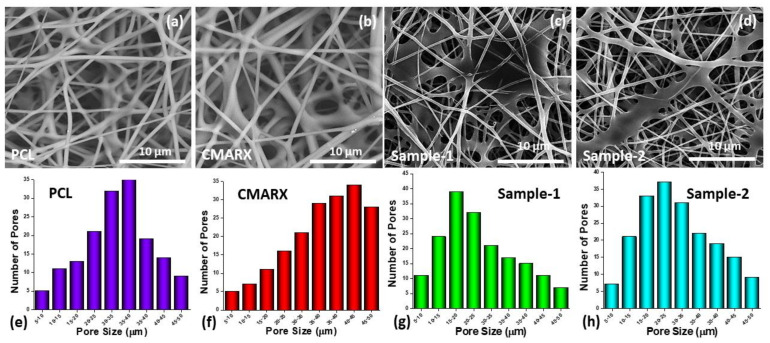
Surface morphology of nanofibrous mat by SEM analysis. The surface morphology (**a**–**d**) and pore size distribution (**e**–**h**) of PCL, CMARX, Sample 1, and Sample 2, respectively.

Figure 2 illustrates the results of the SEM study on the surface morphology of recently developed nanofibrous mats. Due to heat-induced crosslinking, the nano-structural behavior was also seen in Samples 1 and 2. The crosslinking and diffusion of nanofibers between one another, as shown in Figure 2c,d, which composite nanomaterials on the fibrous mat may cause, can be used to detect this behavior. Their nanofibers also have a smaller pore size, which may be caused by the extensive crosslinking and composite nanomaterials (Figure 2g,h). Smooth nanofibrous structures can be found in the PCL and CMARX-derived nanofibers. They do not exhibit clustering because there is no esterification-based crosslinking between −OH and −COOH, as shown in Figure 2a,b. They have uniform fiber diameters with uniform porosity of 320–375 nm for PCL and 375–450 nm for CMARX. However, the different diameters and porosity of the nanofibrous mats are because of the different compositions, chemical integrations, and successful fabrication of nanofibers [39]. Sample 2’s pore size makes it ideal for water treatment and eliminating dyes, heavy metals, and other undesirable inorganic anions. Compared to PCL and CMARX, the composite mat (Sample 2) displayed more dense nanofiber networking, making it a superior fibrous mat for wastewater treatment. The elemental analysis of the Sample-1 and Sample-2 have been provided in the Supplementary Figure S4.

### 4.3. Wetting Analysis

**Figure 3:**
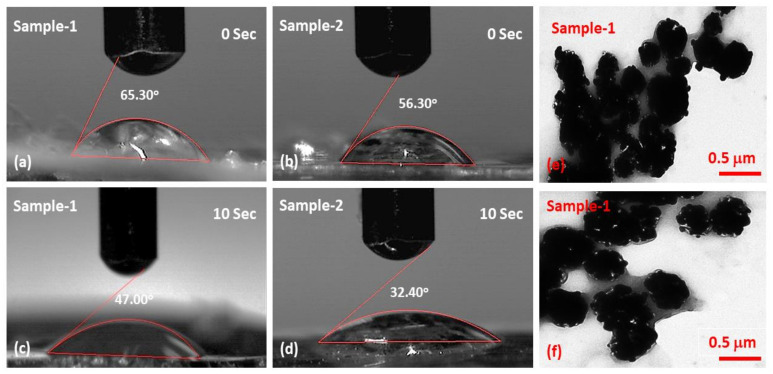
Wetting analysis (**a**–**d**) nanofibrous mat to determine hydrophobicity and hydrophilicity at different intervals (0, 10 s). The TEM analysis (**e**,**f**) of the silver nanoparticles from different areas to see the aggregate.

The wetting behavior is essential for interacting with water and determining hydrophilicity and hydrophobicity in order to understand the composite nanofibrous mats. In general, a material’s hydrophilic nature is represented by a lower contact angle, while a larger contact angle represents its hydrophobic nature. The various oxygen-based functional groups in the composite nanofibrous mats provide them with hydrophilic properties through hydrogen bonding [40]. The reduction in the water contact angle was observed by increasing contact time (0 to 10 s) and shifting the hydrophilic behavior towards a more hydrophilic nature, as shown in Figure 3a–d. It is worth mentioning that Sample 2 (65.30°@0 s and 47.00°@10 s) is more hydrophilic than Sample 1 (56.30°@0 s and 32.40°@10 s), which may be due to the incorporation of silver nanoparticles. Wetting behavior is essential for wastewater treatment because it enhances the properties of materials and surface activities [41]. Hydrophilic behavior is crucial for wastewater treatment because it enables water to interact with composite nanofibrous and remove unwanted materials or pollutants.

### 4.4. TEM Analysis

Figure 3e,f displays the TEM morphology of the mounted nanofibrous silver nanoparticles. The silver nanoparticles seemed to start aggregating after one hour. Given that silver nanoparticles were produced in an aqueous solution and had a variety of hydroxyl functional groups on their surface. Because the silver nanoparticles’ microenvironment in the aqueous solution was neutral, hydrogen bonds was able to form there [42]. Nanofibrous mats can bind many CMARX molecules because they have numerous additional carboxyl groups on their surface. Therefore, silver nanoparticles with numerous hydroxyl groups can easily anchor and aggregate onto the surface of generated nanofibers, primarily due to hydrogen bonding.

### 4.5. Mechanical Testing

**Figure 4:**
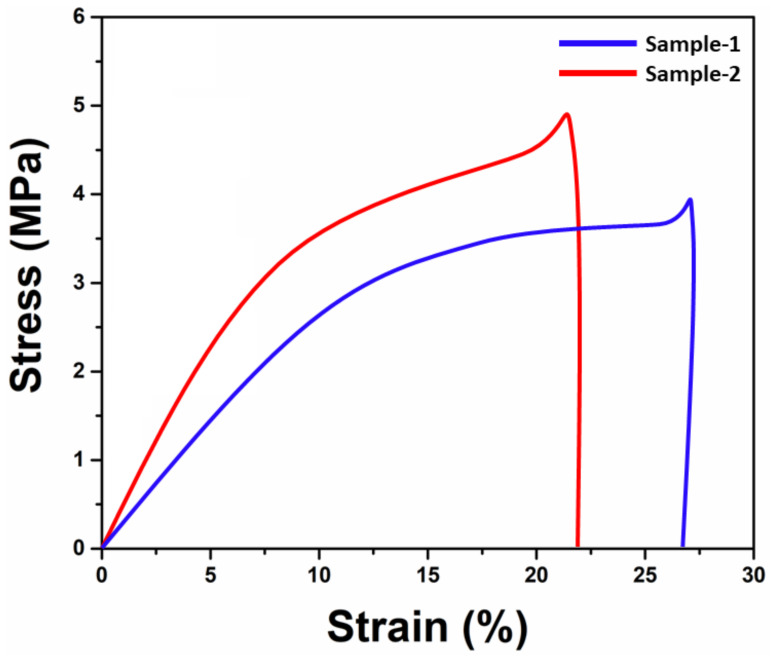
Stress–strain curve to determine the mechanical behavior of the nanofibrous mats.

It has been found that the electrospun nano-fibrous mats’ mechanical properties are crucial for treating wastewater. Figure 4 shows the findings of a stress–strain curve investigation into the mechanical behavior of Sample 1 and blended Sample 2. While Sample 2 displayed the least strain and the highest tensile strength, Sample 1 displayed a stronger strain and a lower tensile strength. These results suggested that adding silver nanoparticles to Sample 2 enhanced its tensile properties. Additionally, it was discovered that Sample 2 behaved differently than Sample 1 because Sample 2 experiences less strain as stress levels rise. However, Sample 1 has less stress values and more strain, possibly due to increasing active or binding sites that increase the crosslinking phenomena [43]. Silver nanoparticles also resulted in Sample 2 having a smaller overall diameter despite a higher surface area. This suggests the presence of many nodes in the fiber pores, which improves the tensile strength behavior.

### 4.6. Adsorption Kinetics

**Figure 5:**
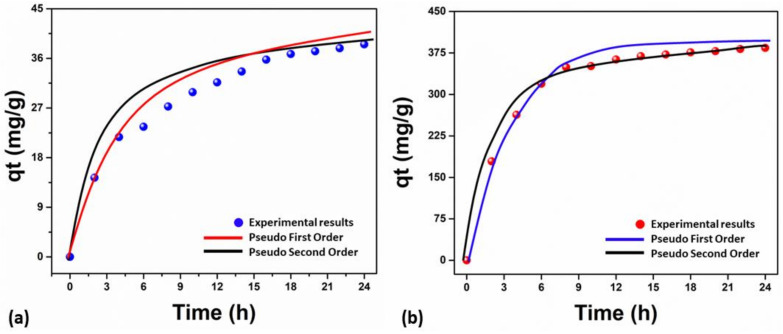
The kinetic absorption analysis of MB uses the nanofibrous mat’s pseudo-first order and pseudo-second order; Sample 1 (**a**), Sample 2 (**b**).

The rate constants for the entire adsorption process were calculated by studying the kinetics of dye adsorption. As shown in Figure 5a,b, the dye contact time with the adsorber was measured to examine the kinetic adsorption behavior of MB for Samples 1 and 2. At first, a quick adsorption rate was seen, but eventually, it reached equilibrium. While other samples (Sample 1 and Sample 2) reached equilibrium for MB adsorption after 18 h and 12 h, respectively, about 78% of MB adsorption was observed within 5 h. The results show that Sample 2 has exhibited higher MB adsorption due to additional active sites and multifunctional, which is possible because of the incorporations of silver nanoparticles [44]. However, the fitting of adsorption kinetics was observed to be non-linear pseudo-first order and pseudo-second order models, as shown in Figure 5a,b. The correlation coefficient of the pseudo-first order (R^2^ = 0.9861) is greater than that of the pseudo-second order (R^2^ = 0.9793) for Sample 1. The correlation coefficient of the pseudo-second order (R^2^ = 0.9861) is higher than that of the pseudo-first order (R^2^ = 0.9793) for Sample 2. However, the correlation coefficient of the pseudo-first order (R^2^ = 0.9917) was lower for Sample 2 and higher for the pseudo-second order (R^2^ = 0.9978) for Sample 1, and these values are summarized in Table 1. These findings imply that the pseudo-second order kinetic model adequately fitted the MB adsorption onto Sample 1 and Sample 2. It was established that charge attraction between adsorbent binding sites and MB occurs during the chemisorption pathway and dominates the adsorption rate of MB.

### 4.7. Adsorption Isotherms

**Figure 6:**
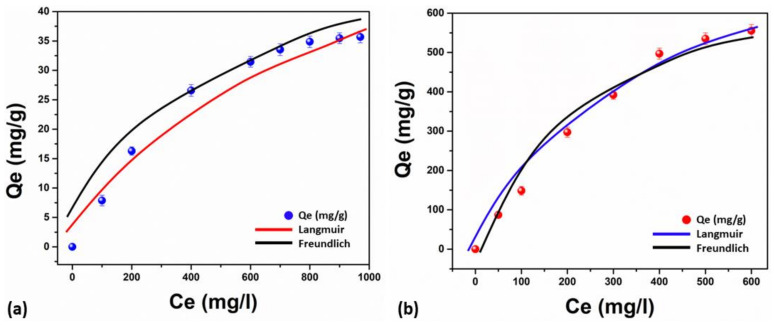
The adsorption isotherms analysis by equations, i.e., the Langmuir and Freundlich models of the nanofibrous mat.; Sample 1 (**a**), Sample 2 (**b**).

The adsorption isotherms contain crucial information about the sorption process. The highest adsorption capacity was determined by this study’s correlations between adsorbent and adsorbate concentrations. Through the use of the well-known non-linear isotherm models, i.e., Langmuir and Freundlich, this study evaluated the performance of adsorption. According to the Langmuir fitted model, the adsorbent’s uniform solid surface was coated with a monolayer as the adsorption mechanism. There is no evidence for the interaction of MB with nearby binding sites. The multi-layer adsorption mechanism on the adsorbent’s heterogeneous solid surface was seen using the Freundlich model, as shown in Figure 6a,b. The calculated isotherm parameters are shown in Table 2. The correlation co-efficiency values (R^2^) demonstrate that these isotherms of adsorption followed the Langmuir isotherm model, which confirms that the monolayer adsorption is linked to the heterogeneous surface of adsorbents. Compared to Sample 1, which had a lower adsorption capacity (53.0472 mg/g), Sample 2 exhibits an increased adsorption capacity (1129.5809 mg/g). This shows that Sample 2 enhanced the adsorption ability of MB, which is integrated with the value of KL, as the adsorbent’s higher KL determines excellent adsorption efficiency at low concentrations. According to Table 2, when our adsorbents were compared to other adsorbent materials for MB adsorption, Sample 2 had a significantly greater ability to adsorb than Sample 1.

### 4.8. Inorganic Anionic Effect

**Figure 7:**
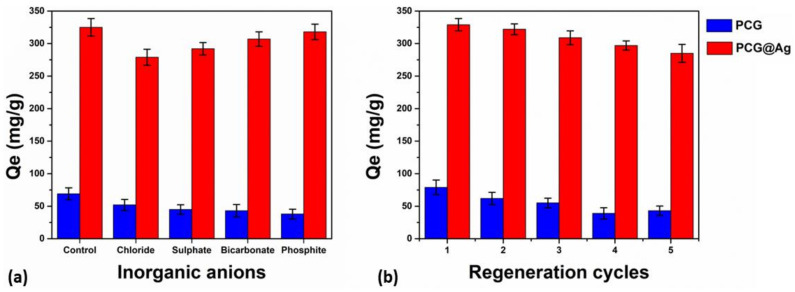
The adsorption capacity of inorganic anions onto MB-absorbed mats (**a**) and the recyclability (**b**) of the nanofibrous mats.

Several inorganic salts have been used to process and manufacture dyes, and several toxic inorganic anions have been found in industrial wastewater [45,46]. Industrial wastewater and effluent are discarded into rivers and freshwater bodies without treatment. Then, these can contaminate and pollute natural water bodies, reducing the availability of drinking water and other utilities [47,48]. Hence, it is important to investigate the anions containing MB-stimulating effluents, and we used inorganic anions (such as Cl^−^, SO_4_^2−^, HCO_3_^−^, and PO_4_^3−^) to achieve this. We investigated their influence on MB adsorption capacity by the mat, as shown in Figure 7a. As expected, the MB was able to adsorb less material when different inorganic anions were present than when they were absent. It was discovered that Cl^−^ ions had a detrimental impact on the mat’s MB adsorption behavior, which could be due to their competition with foreign anions. The chloride anions are more reactive to be adsorbed than the positive dye. However, anions have a lower binding constant to MB, and SO_4_^2−^ and HCO_3_^−^ had little impact on the mat’s ability to adsorb MB. It is interesting to note that the availability of hydrogen phosphate ions increased the pH of the solution, which in turn enhanced the adsorption capability after the response of PO_4_^3−^. As a result, there may be an acceleration of the electrodynamic activity between the mat and the MB adsorption, which would improve adsorption efficiency.

### 4.9. Reusability

Compared to other nanofibrous mats (Sample 1), the composite nanofibrous mat (Sample 2) has shown greater adsorption and reusability. The results of the newly developed composite nanofibrous mat revealed it to be an efficient absorbent to treat wastewater [49]. The composite nanofibrous mats were tested for several regeneration cycles, and under optimal conditions, these composite nanofibrous mats lasted up to five cycles, as shown in Figure 7b. Moreover, a 29% and 0.86% reducing adsorption capacity was observed after five cycles for Sample 1 and Sample 2, respectively, which confirmed that Sample 2 had enhanced reusability compared to Sample 1. The exceptional reusability of Sample 2 may result from its multifunctional behavior, which provides several active sites for adsorption, growing surface areas, and hydrophilic behavior with increasing contact time (as demonstrated in Figure 3a–d). [50]. Therefore, it is confirmed that the Sample 2 composite nanofibrous mat could be a potential adsorption material for wastewater treatment with reusability.

## 5. Conclusions

The composite nanofibrous mat is fabricated from PCL, GO, and silver nanoparticles to remove the dyes to treat wastewater treatment with excellent properties. Sample 2 has an exceptionally smaller diameter than the PCL nanofiber mat, according to the analysis of the SEM morphology. Additionally, increasing the mechanical stability and hydrophilicity of Sample 2 and silver nanoparticles causes them to become more hydrophilic (with a contact angle of 56.30° to 32.40°), effectively increasing MB adsorption. Adsorption kinetics confirmed that the pseudo-second order model has a maximum value to linear correlation coefficient (R^2^ = 0.9978) than the pseudo-first order model. However, the adsorption equilibrium was reached after 12 h for Sample 2 and 18 h for Sample 1, respectively. Moreover, the adsorption isotherm confirms that Sample 2 has an exceptional ability to remove MB (1129.5809 mg/g) under normal conditions in comparison to Sample 1 (48 mg/g), and it also follows the Langmuir model. However, the inorganic anion PO_4_^3−^ has more enhanced adsorption than other anions (including Cl^−^, SO_4_^2−^, and HCO_3_^−^). Sample 2 has exhibited exceptional recyclability during up to 5 cycles with improved adsorption capacity. Hence, all the findings support the confirmation that Sample 2 is the best sample and has the ability to treat wastewater, including organic and inorganic pollutants. As a result, Sample 2 is a promising, environmentally friendly, and long-lasting nanofibrous adsorbent for wastewater treatment.

## Figures and Tables

**Table 1:** The kinetic adsorption studies of MB using Sample 1 and Sample 2 are summarized.

Nanofibrous Mat	Pseudo-First Order			Pseudo-Second Order		
	K_1_ (min^−1^)	qe (mg/g)	R^2^	K_2_ (g/mg min)	qe (mg/g)	R^2^
**Sample 1**	0.0048	31.3852	0.9793	0.0002	37.5372	0.9861
**Sample 2**	0.4094	418.6793	0.9917	0.001	498.6849	0.9978

**Table 2:** MB adsorption and its isotherm parameters.

Nanofibrous Mat	Langmuir			Freundlich		
	q_max_ (mg/g)	K_L_ (L/mg)	R^2^	K_F_ (mg/g)	1/n	R^2^
**Sample 1**	53.0472	0.0034	0.9872	1.0152	0.4739	0.9731
**Sample 2**	1129.5809	0.0437	0.9939	15.0093	0.7164	0.9864

## Data Availability

The data is contained in the manuscript.

## Supplementary Materials

The following supporting information can be downloaded at: https://www.mdpi.com/article/10.3390/membranes13070622/s1, Figure S1: FTIR of PLA; Figure S2: XRD of PCG; Figure S3: XRD of PCG@Ag; Figure S4: SEM and EDX of sample-1 and sample-2.
