# Supplementary material for: Fabrication of PCL/CMARX/GO Composite Nanofibrous Mats for Dye Adsorption: Wastewater Treatment
Source: Membranes (Basel). 2023 Jun 26;13(7):622. doi: 10.3390/membranes13070622 (PMC10383201; doi:10.3390/membranes13070622)
Supplement: Supplementary file 1 [file membranes-13-00622-s001.zip › membranes-2395940-supplementary.pdf]

Supporting information

Fabrication of PCL/CMARX/GO Composite Nanofibrous Mats for Dye Adsorption: Wastewater Treatment

Mona Saad Binkadem *

Department of Chemistry, college of science, University of Jeddah, P.O. Box 80327, Jeddah 21589, Saudi Arabia.

* Correspondence: 04100507@uj.edu.sa

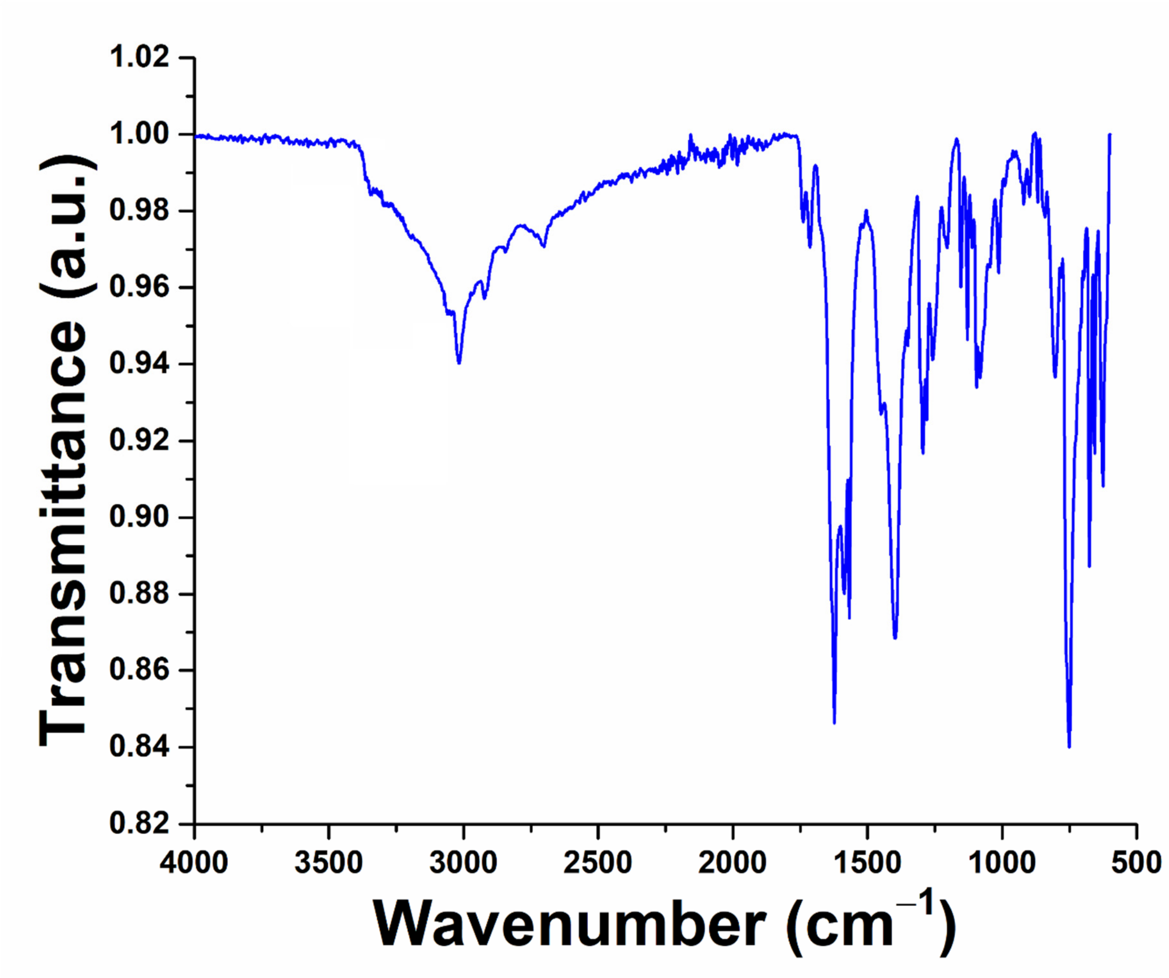

Figure S1. FTIR spectrum of PLA.

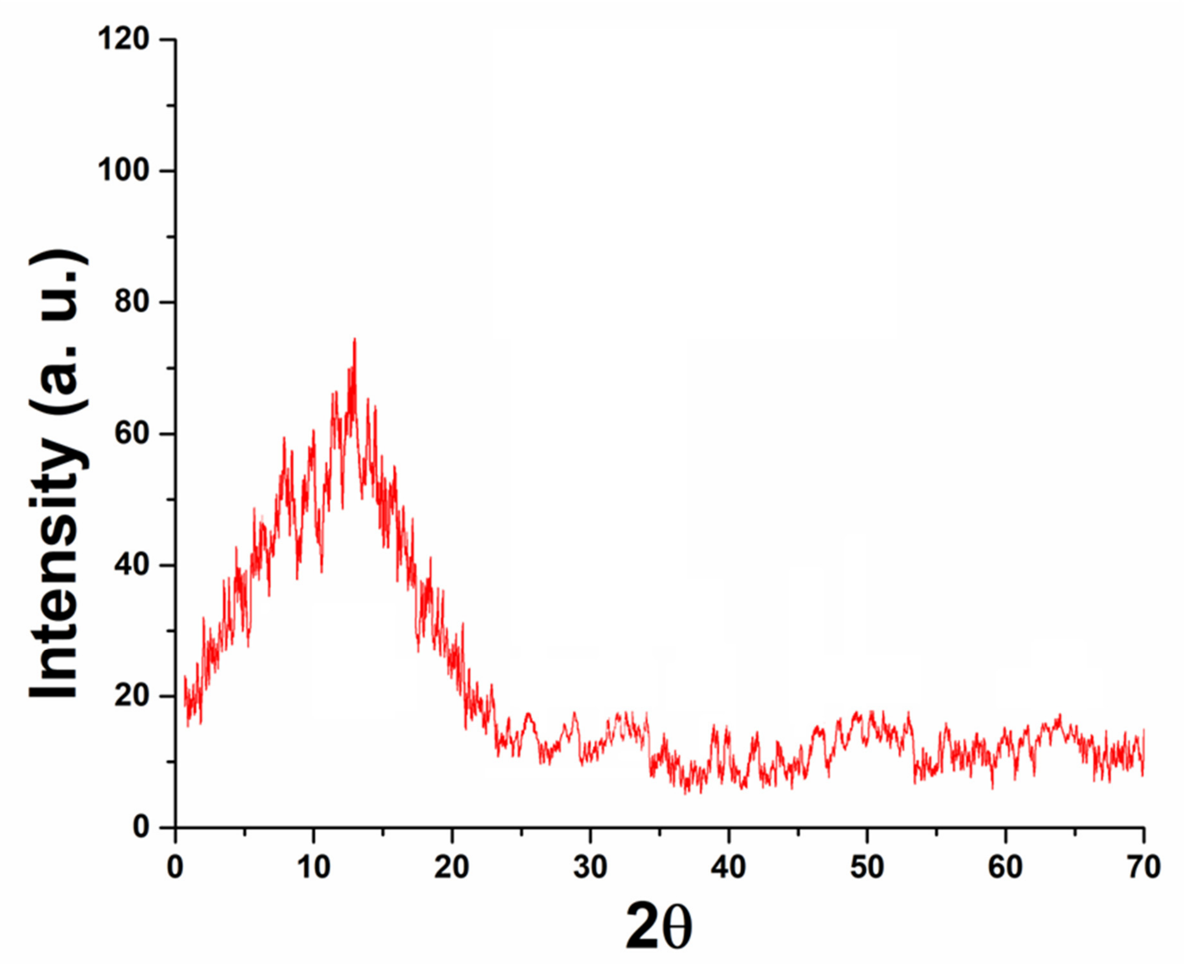

Figure S2. XRD spectrum of PCG.

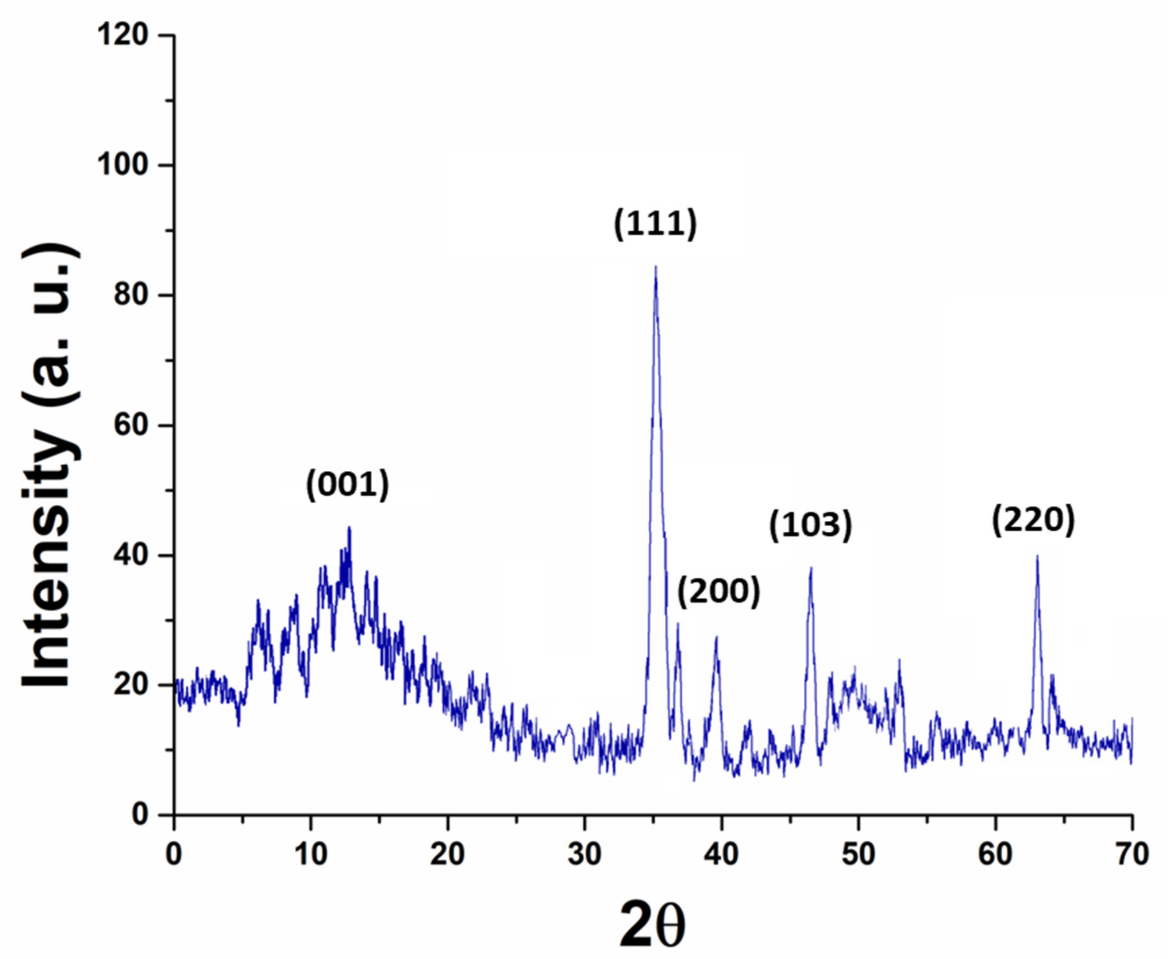

Figure S3. XRD spectrum of PCG@Ag.

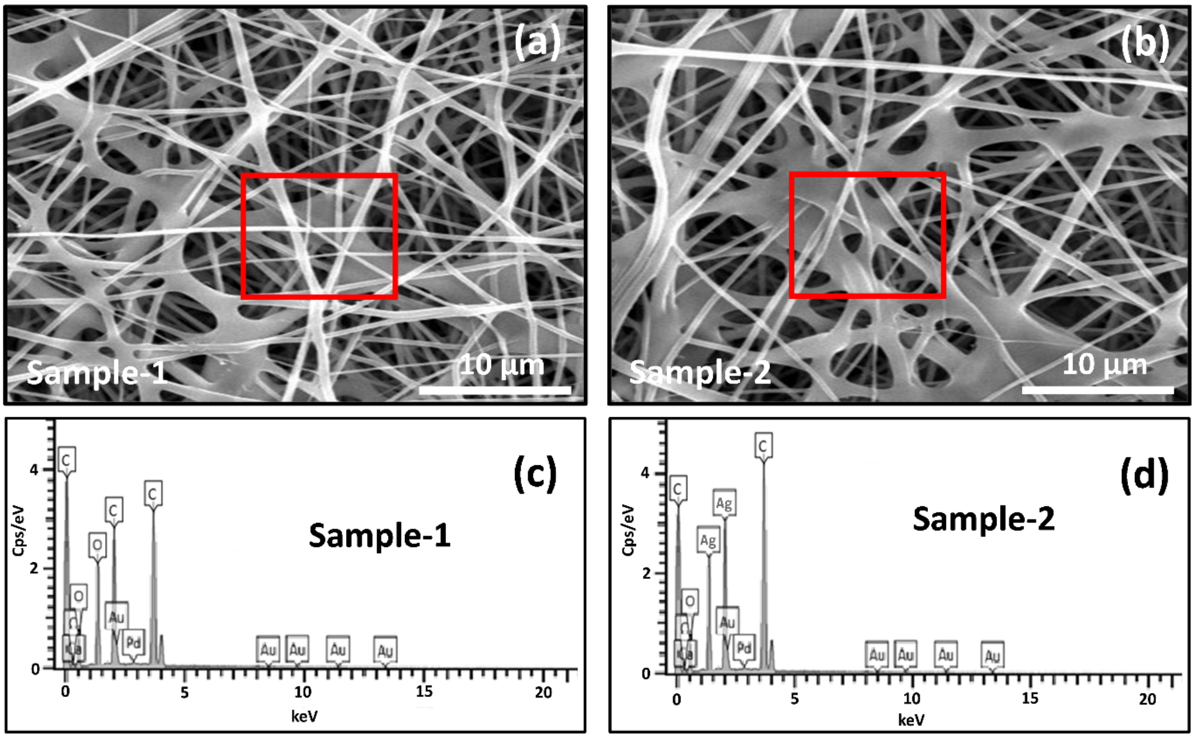

Element	Weight (%)	Weight (%)
C	43.52	54.19
H	24.61	21.03
O	31.87	24.78
Ag	0	0

Element	Weight (%)	Weight (%)
C	36.94	41.83
H	19.82	13.69
O	28.56	23.49
Ag	14.68	20.99

Figure S4. Surface morphologies and elemental analysis of the nanofibrous membranes (sample-1 and sample-2) by single electron microscope (SEM) and Energy Dispersive X-ray (EDX) microanalysis. (a and b) SEM analysis of sample-1 and sample-2 at 10 µm. (c and d) EDX analysis of sample-1 and sample-2 alongwith their elemental profile.
